# AAV‐Mediated Expression of Methamphetamine Monoclonal Antibody Attenuates Methamphetamine Behaviour Sensitization in Mice

**DOI:** 10.1111/adb.70073

**Published:** 2025-08-08

**Authors:** Yun‐Hsiang Chen, Tsai‐Wei Hung, Yu‐Syuan Wang, Eun‐Kyung Bae, Kuo‐Jen Wu, Yun Wang, Seong‐Jin Yu

**Affiliations:** ^1^ Center for Neuropsychiatric Research National Health Research Institutes Zhunan Taiwan; ^2^ Department of Life Science Fu‐Jen Catholic University New Taipei City Taiwan; ^3^ School of Pharmacy, College of Pharmacy China Medical University Taichung Taiwan

**Keywords:** adeno‐associated virus, antibody therapy, methamphetamine, sensitization

## Abstract

Methamphetamine (Meth) is a psychoactive and neurotoxic chemical. Selective antibodies against Meth molecules have been examined for the treatment of Meth abuse through immunization. Antibodies with high affinity for Meth can capture Meth molecules and reduce Meth response. We previously reported that intraperitoneal administration of adeno‐associated virus serotype vector serotype 8 carrying Meth‐specific monoclonal antibody transgene (AAV8‐MethAb, 2.5 × 10^10^ VGC per mouse) induced long‐term and stable expression of Meth‐antibody in the peripheral. Mice receiving AAV8‐MethAb had a lower Meth level in the blood and brain and attenuated Meth‐induced locomotor activity after an acute dose of Meth. The effect of AAV‐MethAb in animals receiving repeated Meth administration was still not known. In this study, we first investigated the tropism of AAV serotypes in rat primary dopaminergic (DA) neuronal culture. We found that AAV6 is an optimal gene carrier for MethAb. AAV6‐MethAb or AAV6‐mCherry was used in cellular and animal models of chronic Meth use. In primary DA neuronal culture, repeated Meth administration increased the dendritic branching of DA neurons, which was antagonized by AAV6‐MethAb. AAV6‐MethAb or AAV6‐mCherry was stereotaxically administered to the nucleus accumbens (NAc) of adult CD1 mice. Two weeks after the viral injection, animals were stimulated with a daily dose of Meth for 7 days. Repeat Meth administrations led to a progressive increase in locomotor activity or behaviour sensitization. This response was significantly attenuated in mice receiving AAV6‐MethAb. Using qRTPCR and Western analysis, we demonstrated that MethAb mRNA and protein were expressed in the NAc. Previous reports indicated that Meth sensitization was associated with upregulation of tyrosine hydroxylase (TH) in the NAc. Using Western blot analysis, we found that AAV6‐MethAb significantly reduced TH protein levels in Meth‐sensitized mice. Taken together, our data support that intracerebral administration of AAV6‐MethAb reduced Meth sensitization. Our data support a novel antibody gene therapy for Meth abuse.

## Graphical abstract

AAV6‐MethAb attenuated Meth‐induced neuro‐sprouting. AAV‐mediated MethAb expression in the nucleus accumbens antagonized Meth‐induced behavioural sensitization, along with attenuating TH expression.

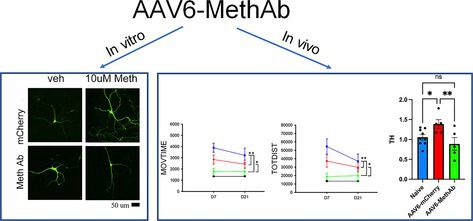

## 1 Introduction

Methamphetamine (Meth) is a highly addictive psychoactive substance. Meth abuse leads to cognitive dysfunction, neurodegeneration and numerous complications and has become a public health and social concern around the world. Currently, there is still no effective medical therapy for Meth addiction.

Meth mediates the release of presynaptic dopamine (DA), activates post‐synaptic DA receptors and alters dopaminergic functions. In response to reward‐related stimuli (e.g., Meth), the dopaminergic neuron projections from the ventral tegmental area (VTA) to the nucleus accumbens (NAc) release DA. Meth also interacts with the proteins involved in the transportation and metabolism of dopamine (DA), leading to the accumulation of dopamine in the extracellular space of dopaminergic terminals in the NAc. These responses are critical for acute rewarding effects and initiation of addiction [1].

Meth sensitization is characterized as a progressive increase in behavioural responses after repeated Meth administration. It has been demonstrated that repeated administration of Meth enhanced the ambulation‐increasing effect in mice [2]. In addition, the development of sensitization during Meth abuse plays a central role in the aetiology of Meth psychosis [3]. At the molecular level, repeated exposure to Meth or its analogues induces substantial changes in neural plasticity, particularly in the nucleus accumbens (NAc), a brain region integral to reward and addiction pathways. These changes involve the generation of nascent synapses mediated by key molecules such as brain‐derived neurotrophic factor (BDNF), cAMP response element‐binding protein (CREB), ΔFosB and other synaptogenesis‐related proteins [4, 5, 6]. Newly grown dendritic spines form these synapses in conjunction with axons sprouting from the presynaptic terminals, creating new neural circuits [7].

Various DA receptor antagonists have been shown to reduce Meth actions, including behavioural sensitization and neurochemical changes [8, 9]. However, DA has differential responses in different brain regions. Chronic Meth users exhibit signs of Parkinson's disease [10]. Treatment with DA antagonists may further enhance Parkinson's symptoms in these patients. We and others previously reported that Meth induces Ca++ influx from non‐dopaminergic neurons in the cerebral cortex [11] and alters DA‐independent gene transcription in the 6‐hydroxydopamine‐lesioned hemi‐parkinsonian rats [12]. These data suggest the actions of Meth in non‐DA neurons. The use of selective and specific DA receptor antagonists for pharmacotherapies is thus insufficient for Meth abuse.

Selective antibodies against Meth molecules have been examined for the treatment of Meth addiction [13, 14]. In active immunization, Meth‐like small molecules linked to immunogenic carriers were used to stimulate the immune system to produce Meth antibodies in hosts [14, 15, 16]. For passive immunization, hosts were infused intravenously with preselected Meth antibodies derived from vaccinated animals or synthetic antibody libraries [17, 18, 19]. Antibodies with high affinity for Meth capture Meth molecules within the circulatory system and reduce their access to the activation sites in the brain since antibodies are too large to readily cross the blood–brain barrier [20, 21]. Therefore, circulating Meth antibodies act as antagonists to attenuate Meth‐induced behaviour without targeting neural components in the brain.

In our previous study, an alternative approach was used to produce Meth antibodies in the host through adeno‐associated virus (AAV) infection [22]. A recombinant AAV serotype‐8 vector (AAV8) was designed to carry an expression cassette encoding both the heavy‐ and light‐chain genes of Meth antibody (MethAb), which were linked by a self‐cleavage 2A sequence. The light‐chain and heavy‐chain genes of the Meth antibody were genetically linked by a coding sequence of the self‐cleaving 2A peptide and cloned in an expression cassette flanked by two inverted‐terminal repeats (ITR) of AAVs [22]. This design was created to overcome the size limitation of AAV packaging and to produce full‐length antibodies effectively. The 2A peptide, adopted from the foot‐and‐mouth disease virus, can induce ribosome skipping at its C‐terminal during translation. This leads to co‐translating two proteins linked by the 2A peptide at similar levels from a single open reading frame [23]. We reported that intraperitoneal administration of AAV8‐MethAb induced long‐term and stable expression of a Meth‐specific monoclonal antibody in the peripheral blood. Mice receiving AAV8‐MethAb had a lower Meth level in the blood and brain and attenuated Meth‐induced locomotor activity after a single dose of Meth administration [22]. These data suggest that peripherally administered AAV8‐MethAb antagonized acute Meth‐mediated behavioural responses. The therapeutic action of AAV‐MethAb in animals receiving repeated Meth administration is still not known.

**FIGURE 1:**
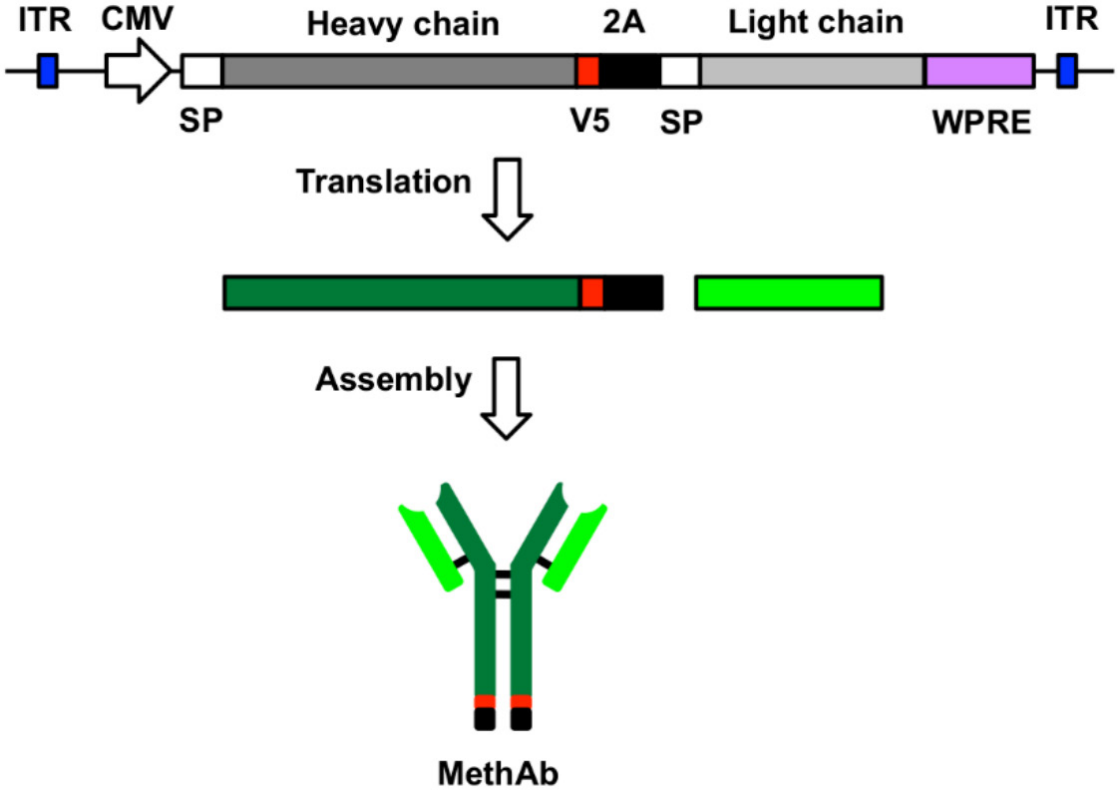
Diagram to illustrate the genetic construct and synthesis of MethAb from AAV‐MethAb. Both heavy‐chain and light‐chain genes of the Meth‐specific antibody (MethAb) were linked by the coding sequence of a 2A self‐cleaving peptide and driven by a cytomegalovirus promoter (CMV) in the expression vector AAV‐MethAb. The 2A peptide can cause ribosome skipping at its C‐terminal, leading to co‐translation of both heavy‐chain and light‐chain of MethAb. MethAb comprises two copies of heavy‐chain and two copies of light‐chain proteins and can be detected by anti‐V5 antibodies in the western blot analysis. ITR: inverted‐terminal repeat of AAV2; SP: signal peptide; V5: V5 tag; WPRE: woodchuck hepatitis B virus post‐transcriptional regulation element.

In this study, AAV vectors of different serotypes (1.2.5.6.7.8) carrying the Meth monoclonal antibody gene were constructed (Figure 1). We found that AAV6 is an optimal gene carrier for expressing MethAb in dopaminergic neurons. We further characterized the response of AAV6‐MethAb in repeated Meth use models in vivo and in vitro. AAV6‐MethAb neutralized Meth‐mediated neuronal sprouting in cultured dopamine neurons. Local administration of AAV6‐MethAb to NAc antagonized Meth sensitization in mice. Our data support a novel antibody gene therapy for Meth abuse.

## 2 Materials and Methods

### 2.1 AAV‐MethAb Virus Plasmids Packaging and Preparation

AAV vectors of serotypes 1.2.5.6.7.8 were constructed to express the Meth monoclonal antibody gene (Figure 1). The HEK 293 cells were seeded at 1.7 × 10^6^ per 15‐cm culture plate in 25 mL of DMEM plus 5% FBS for 4 days before transfection. Subconfluent (70%–80%) monolayer cells were co‐transfected with pAAV‐MethAb (9 μg), RC1.2.5.6.7.8 (3.5 μg) and pHelper (12.5 μg; Agilent Technologies, California, USA) by the TransIT‐VirusGEN Transfection Reagent (Cat. No. MR‐MIR 6700, Mirus, Madison, WI USA). The plasmids used for transfection were purified from *E. coli* . Approximately 54 h post‐transfection, cells were harvested, and the supernatants were mixed with 40% PEG8000 (in 0.9% NaCl) to a final concentration of 8% and incubated at 4°C overnight. Cells were lysed by freeze/thaw; the supernatant mixture was centrifuged at 4000 × *g* at 4°C for 1 h, and the obtained pellet was dissolved in 25‐mL suspension buffer. The virus was purified using the AVB column (28‐4112‐11, GE Healthcare, Chicago, IL USA) and was stored at −80°C until use.

### 2.2 Viral Titres Determination by the Quantitative Real‐Time PCR Assay (qPCR)

Quantitative real‐time PCR was performed on an ABI StepOnePlus system. Five microlitres of the viral sample were pretreated with two units of DNase I (Cat. No. M0303, New England BioLabs, Ipswich, MA, USA) in a final volume of 50 μL at 37°C for 1 h, and then DNase I was inactivated at 75°C for 10 min. The primers that target WPRE on the vector plasmid (pAAV‐MethAb) were used to amplify the qPCR product. Each reaction mixture (20 μL) contained 2 μL of DNase‐pretreated viral sample, 10 μL of 2 × SYBR green PCR master mix (Cat. No. K0371, Thermo, Waltham, MA, USA) and 0.5 μM of each primer. The PCR cycling program was set as the following: 95°C for 10 min followed by 40 cycles for amplification (95°C for 15 s and 60°C for 30 s) and a cycle for generating a melting curve (95°C for 15 s, 60°C for 1 min and 95°C for 15 s). A standard curve using a 10‐fold serial dilution (0.01–100 pg) of the vector plasmid (pAAV‐eGFP) was generated in every qPCR assay. Viral doses were expressed as viral genome copies (VGC) of the virus sample.

### 2.3 Animals

Adult male CD‐1 mice and timed‐pregnant Sprague–Dawley rats were purchased from Lasco, Taiwan. The use of animals and procedures in this study was approved by the Institutional Animal Care and Use Committees of the National Health Research Institutes (approval number: NHRI‐IACUC‐112149‐A). The care and use of animals were conducted in accordance with regulations outlined by the Association for Assessment and Accreditation of Laboratory Animal Care (AAALAC). Animals were housed in a 12‐h dark (7 pm to 7 am) and 12‐h light (7 am to 7 pm) cycle.

### 2.4 Primary Cultures of Rat Ventral Mesencephalon for AAV Serotype Study

Primary dopaminergic neuronal cultures were prepared from embryonic (E15) ventral mesencephalon (VM) tissues obtained from foetuses of timed‐pregnant Sprague–Dawley rats, according to published procedures with some modifications. These cells are commonly used in in vitro drug addiction experiments due to their crucial role in the reward system. The whole brain was removed aseptically, and a small piece of tissue comprising the VM was dissected. After removing the blood vessels and meninges, pooled VM tissues were trypsinized (0.25%; Invitrogen, Carlsbad, CA) with gentle mixing for 15 min at 37°C. After rinsing off trypsin with pre‐warmed DMEM/F‐12 (Invitrogen), cells were dissociated by trituration, counted and plated into 96‐well (6.0 × 10^4^/well) cell culture plates pre‐coated with poly‐D‐lysine (Sigma‐Aldrich). The culture plating medium consisted of Dulbecco's modified Eagle medium/F12 supplemented with 10% heat‐inactivated foetal bovine serum, 1‐mM L‐glutamine and 2% B27 (Invitrogen). Cultures were maintained at 37°C in a humidified atmosphere of 5% CO_2_ and 95% air. The cultures were fed by exchanging 50% of media with feed media (Neurobasal medium, Invitrogen) containing 0.5‐mM L‐glutamate and 2% B27 with antioxidants supplement on DIV (days in vitro) 3 and 5. Various serotypes of AAV viral vectors carrying a gene encoding MethAb were added to the culture on DIV 5 and were fed with media containing B27 supplement without antioxidants (Invitrogen) on DIV7 and 10. Cells were fixed with 4% paraformaldehyde (PFA) for immunoreactivity on DIV12.

### 2.5 Neuronal Culture on Slides

Glass coverslips (18 mm diameter) were placed in 12‐well cell culture plates and coated with poly‐D‐lysine (Sigma‐Aldrich). Primary VM cells (1 × 10^5^ cells/well) were plated into 12‐well plates on DIV0. Viral transductions were performed on DIV 5. On DIV7, cultures were fed with media containing B27 supplements without antioxidants (Invitrogen). Freshly made Meth or saline was added to the wells on DIV 10, 12 and 14 with media containing B27 supplements without antioxidants (Invitrogen). Cells were returned to a 37°C incubator for 24 h and then fixed with 4% paraformaldehyde (PFA). Coverslips were stained with a mouse monoclonal antibody against TH (1:500; Millipore, Billerica, MA) and AlexaFluor 488 goat anti‐mouse secondary (Invitrogen). Images were acquired using a confocal laser‐scanning microscope with a 20× objective and Nikon Eclipse 80i with EZ‐C1 3.9 software.

### 2.6 Intracerebral Injection of AAV6‐mCherry or AAV6‐MethAb in Mice

Mice were anaesthetised with pentobarbital (50 mg/kg, i.p.) and placed in a stereotaxic frame. AAV6‐mCherry (2.5 × 10^8^ VGC/μL × 2 sides; VGC: viral genome copy) or AAV6‐MethAb (2.5 × 10^8^ VGC/μL × 2 sides) was injected into both sides of the nucleus accumbens over 4 min through a 10‐μL Hamilton microsyringe. The tip of microsyringe was moved to the bilateral NAc (coordinate: AP + 1.15 mm, ML ± 1 mm, DV 4.5 mm below skull, according to Paxinos and Franklin's ‘the Mouse Brain’) using micromanipulators attached to the stereotaxic frame. The speed of injection (0.5 μL/min) was controlled by a syringe pump (Micro 4, WPI, Sarasota, FL). The needle was removed 5 min after the injection. A piece of bone wax was placed on the burr hole to prevent the leakage of fluid. The wound was sutured or clipped. Body temperature was monitored with a thermistor probe and maintained at 37°C with a heating pad during anaesthesia. After recovery from the anaesthesia, body temperature was maintained at 37°C for 2 h using a temperature‐controlled incubator.

### 2.7 Meth‐Sensitization and Behaviour Test

Meth behavioural sensitization was conducted as previously described [24]. Mice were treated with Meth (2.5 mg/kg/day, s.c.) or saline for 7 days. On the day of the behaviour test, animals were first placed into locomotor chambers (Accuscan, Columbus, Ohio, USA) for 1‐h acclimatization. Locomotor activity was recorded for 1 h after injection of Meth (1 mg/kg, s.c.). Analysis was conducted using the total distance travelled (TOTDIST; the distance travelled in centimetres), horizontal activity (HACTV; the total number of beam interruptions that occurred in the horizontal sensors), movement time (MOVTIME, the amount of time in ambulation) and stereotypic movement number (STRCNT).

### 2.8 Western Blot

The NAc tissues were harvested and homogenized in RIPA lysis buffer (Merck Millipore, Burlington, MA, USA) and centrifuged at 13 200 rpm for 10 min at 4°C. The supernatant was collected. A bicinchoninic acid (BCA) protein assay was performed to determine protein concentrations. The samples were diluted according to the BCA protein assay. Gels were transferred to a PVDF membrane after electrophoresis. The membranes were blocked in 5% milk at room temp for 1 h. The blots were then probed with primary antibodies against tyrosine hydroxylase (polyclonal, TH, 1: 10 000, Millipore, Burlington, MA, USA) at 4°C overnight. The membrane was then incubated with horseradish peroxidase (HRP)‐conjugated secondary antibody (Jackson lab, Bar Harbor, Maine, USA) at room temp for 1 h, followed by washing with 0.1% Tween‐20 (in PBS) three times for 10 min each. The light emission signal of the target proteins on the PVDF membrane was generated by using a Western Lightning Plus‐ECL (PerkinElmer, Waltham, MA, USA) and then detected by X‐ray film (Cat. No. GE28‐9068‐39, GE, Boston, MA, USA). The immunoreactivity of TH was normalized with actin on the same membrane. Band intensity was quantified using Image J.

### 2.9 Quantitative Reverse Transcription PCR (qRTPCR)

Cells or brain tissues were collected for qRT‐PCR analysis. Total RNAs were isolated using TRIzol Reagent (ThermoFisher, #15596‐018, Waltham, MA, USA), and cDNAs were synthesized from 1‐μg total RNA using RevertAid H Minus First Strand cDNA Synthesis Kit (ThermoFisher, #K1631, Waltham, MA, USA). cDNA levels for 6H4 HV (MethAb), mCherry and GAPDH were determined by specific universal probe Library primer‐probe sets or gene‐specific primers (Table 1). Quantitative real‐time PCR (qRT‐PCR) was carried out using TaqMan Fast Advanced Master Mix (Applied Biosystems, #4444557, Waltham, MA, USA) or SYBR (Luminaris Color HiGreen Low ROX qPCR Master Mix; ThermoScientific) and the QuantStudio 3 Real‐Time PCR System (ThermoScientific, Waltham, MA, USA). The expression of the target genes was normalized relative to the endogenous reference gene (GAPDH) with a modified delta–delta‐Ct algorithm. All experiments were carried out in duplicate.

**TABLE 1:** Oligonucleotide primers used for quantitative RT‐PCR.

	SYBR green		TagMan
Gene	Forward	Reverse	
6H4 HV	GCCTCGTGAAACCTTCTCAG	CCGAATCCAGCTCCAGTAAC	
mCherry	CCTGTCCCCTCAGTTCATGT	CCCATGGTCTTCTTCTGCAT	
GAPDH			Mm99999915_g1

### 2.10 Statistical Analysis

Data were presented as mean ± SEM. Student's *t*‐test, one or two‐way ANOVA and post hoc Fisher LSD test were used for statistical comparisons. Statistical significance was defined as *p* < 0.05.

## 3 Results

### 3.1 The Expression of MethAb in Dopaminergic Neuronal Culture

**FIGURE 2:**
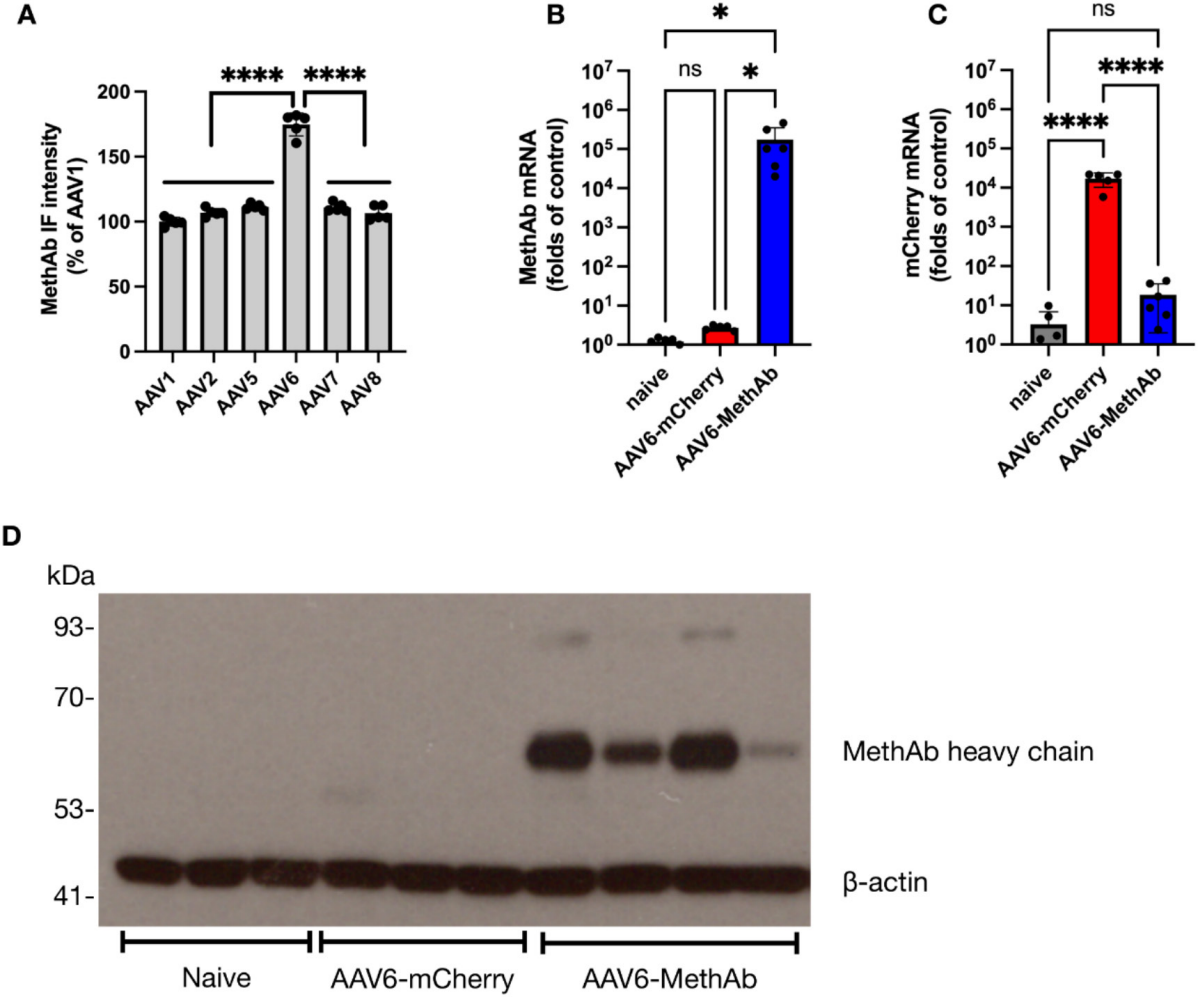
Effects of viral serotypes on the expression of MethAb in primary VM cell culture and in vivo. On day 5 of in vitro, rat VM cells were transduced with various serotypes of AAV‐MethAb viral vectors (AAV1, AAV2, AAV5, AAV6, AAV7 and AAV8). On day 15 of in vitro, cells were subjected to immunofluorescence staining to measure the expression of MethAb. (A) The integrated fluorescence density (IF) was calculated from images captured from five microscopic fields and normalized to the AAV1‐transduced group. (B, C) Mice were intracerebral (NAc) injected with AAV6‐MethAb or AAV6‐mCherry. NAc tissues were collected to quantify (B) MethAb mRNA and (C) mCherry mRNA by qRT‐PCR. (B) MethAb mRNA was detected only in the animals receiving AAV6‐MethAb. (C) The expression of mCherry mRNA was significantly increased in mice injected with AAV6‐mCherry. The mRNA levels were normalized to GAPDH and expressed as fold changes relative to the control (naive animal) using the 2‐∆∆Ct method. (D) NAc tissues were collected for Western blot analysis to detect the heavy chain of MethAb using anti‐V5 tag antibodies. Equal sample loading was confirmed by probing β‐actin. MethAb was only detected in mice injected with AAV6‐MethAb. Naive: rats were not injected with any viral vector. ****p < 0.0001, *p < 0.05; ns: not significant.

To investigate the tropism of AAV serotypes on the expression of MethAb, we transduced cultured rat dopaminergic cells with various serotypes of AAV viral vectors (AAV1, AAV2, AAV5, AAV6, AAV7 and AAV8) carrying the MethAb transgene. Ten days after viral transduction, the cells were fixed and subjected to immunofluorescence staining to detect MethAb. The AAV6 transduced a significantly higher level of MethAb than the other AAV serotypes (Figure 2A: AAV6: 174.8% ± 3.9; AAV1: 100% ± 1.5, *p* < 0.0001; AAV2: 107% ± 1.4, *p* < 0.0001; AAV5: 111.4% ± 1.1, *p* < 0.0001; AAV7: 110.9% ± 1.5, *p* < 0.0001; AAV8: 106.6% ± 2.4, *p* < 0.0001). These results suggest that AAV6 is an optimal gene carrier for MethAb in dopaminergic neurons.

### 3.2 The Expression of MethAb and mCherry in Mouse Brain

AAV6‐MethAb or AAV6‐mCherry were administered stereotaxically to the NAc of adult mice. Brain tissues (NAc) were collected 23 days after viral injection. The mRNAs of MethAb and mCherry were probed by gene‐specific primers and quantified by qRT‐PCR. MethAb mRNA was readily detected only in the animals receiving AAV6‐MethAb (Figure 2B, *p* = 0.013), but not AAV6‐mCherry or the naïve. Similarly, the expression of mCherry mRNA was significantly increased in mice injected with AAV6‐mCherry (*p* < 0.0001, Figure 2C). Using Western blot analysis, MethAb was only detected in mice receiving AAV6‐MethAb (Figure 2D). These data indicate that AAV6 is suitable for gene delivery to express MethAb in the mouse brain.

### 3.3 AAV6‐MethAb Antagonized Meth‐Induced Neuronal Branching in Cultured Dopaminergic Neurons

**FIGURE 3:**
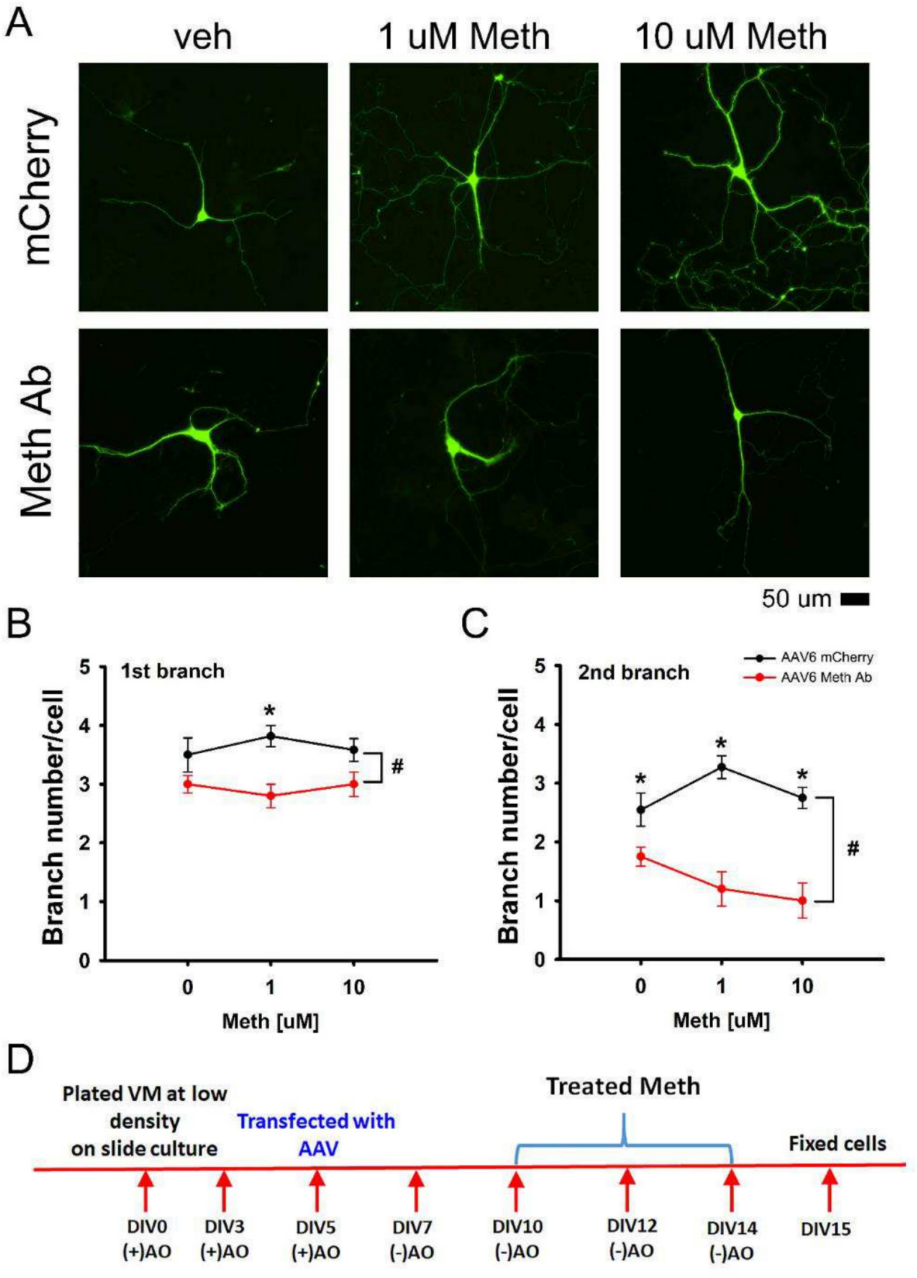
AAV6‐MethAb reduced Meth‐mediated dendritic branching of cultured dopaminergic neurons. (D) Timeline. Primary dopaminergic cells were transfected with AAV6‐mCherry or AAV6‐MethAb on DIV5 and were treated with Meth or vehicle on DIV 10, 12 and 14. TH immunoreactivity was examined on DIV15. (A) Confocal photomicrographs showed that low dose (1 or 10 μM) Meth administration increased the dendritic branching of the dopaminergic neurons in the control cells transducing with AAV6‐mCherry (upper panels). AAV6‐MethAb antagonized this response (lower panels). (B) The number of dendritic branches was measured from AAV6‐mCherry (veh, *n* = 12; 1 μM, *n* = 11; 10 μM, *n* = 12) or AAV6‐MethAb‐treated groups (*n* = 10, each). AAV6‐MethAb significantly reduced primary (*p* < 0.001, two‐way ANOVA) and secondary branch numbers (*p* < 0.001, two‐way ANOVA). Scale: 50 μm. (+)AO: culture media with antioxidants. (−)AO: culture media without antioxidants.

Primary dopaminergic neuronal cells prepared from embryonic (E15) ventral mesencephalon (VM) tissues were cultured on slide chambers and transfected with AAV6‐mCherry or AAV6‐MethAb on DIV5 (see timeline, Figure 3D). Cells were repeatedly treated with Meth or vehicle on DIV 10, 12 and 14. They were fixed for TH immunocytochemistry on DIV15. The branching of TH cells was counted using a confocal microscope. As seen in Figure 3A (upper panels), repeated low‐dose (1 or 10 μM) Meth administration increased the branching of dopamine neurons receiving AAV6‐mCherry. In contrast, AAV‐MethAb reduced Meth‐mediated branching (Figure 3A, lower panels).

The primary and secondary dendritic branches were further analysed from 127 dopaminergic neurons. Meth significantly increased the number of primary (Figure 2B) and secondary branches (Figure 3C). AAV6‐MethAb significantly reduced the Meth‐mediated spouting (*p* < 0.001, 2‐way ANOVA, Figure 3B,C).

### 3.4 Intracerebral Injection of AAV6‐MethAb Reduces Behavioural Sensitization

**FIGURE 4:**
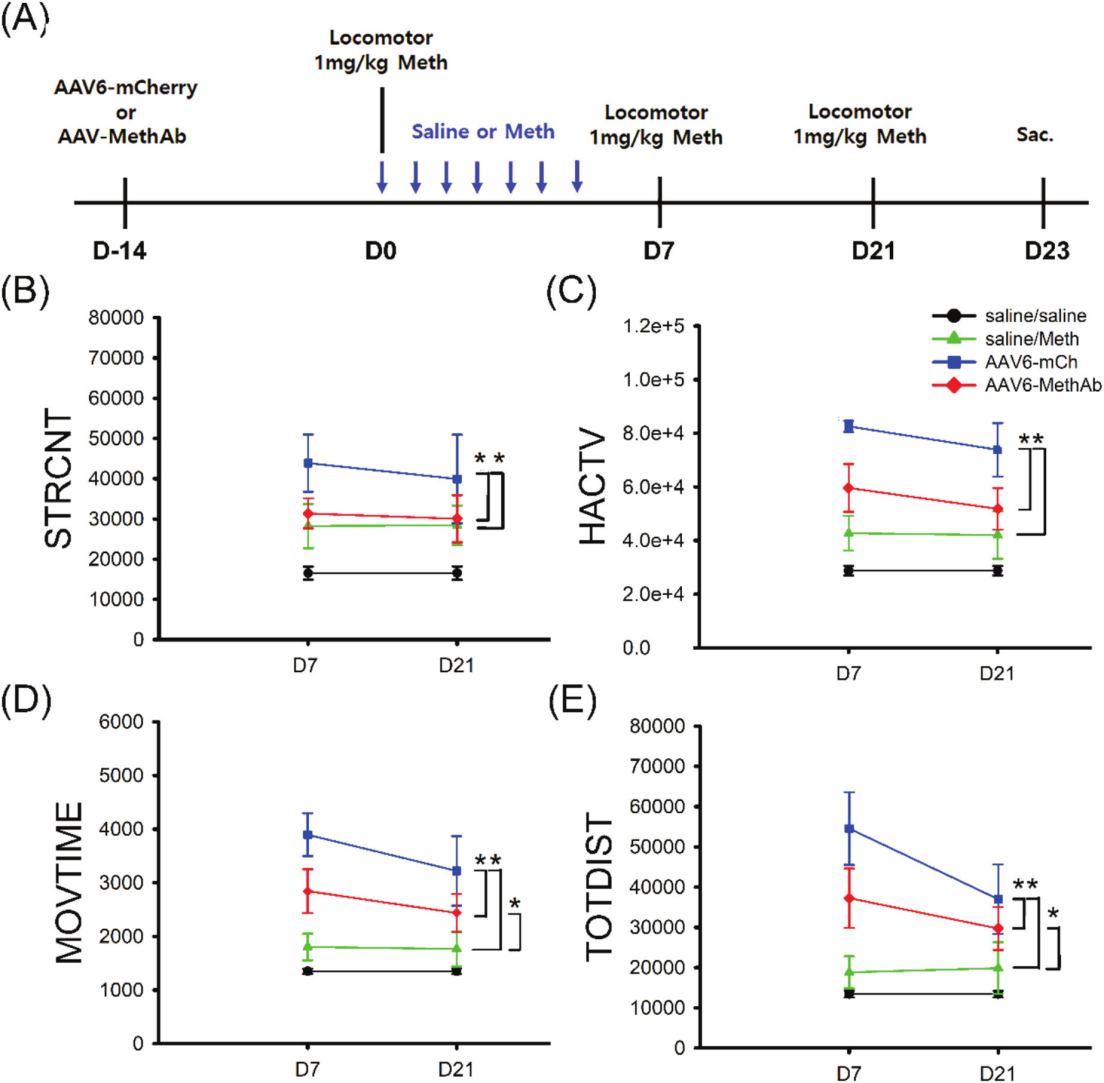
Intracerebral administration of AAV6‐Meth Ab reduced Meth‐mediated behavioural sensitization. (A) Timeline. Adult mice received bilateral NAc administration of AAV6‐mCherry (2.5 × 10^8^ VGC/μL × 2 sides; VGC: viral genome copy) or AAV6‐Meth Ab (2.5 × 10^8^ VGC/μL × 2 sides) 14 days (D‐14) before the induction of Meth sensitization. A daily dose of Meth (2.5 mg/kg/day) was given to these animals from day 0 to day 6. Control animals (saline/saline and saline/Meth) received daily saline injection (7 days, D0–D6). Meth (1 mg/kg, s.c.) induced locomotor activity was recorded on days 7 and 21 in animals receiving AAV6‐mCherry or AAV6‐MethAb, and saline/Meth. Saline/saline group animals were given saline on the test days (D7 and 21). (B–E) Repeated Meth treatment (AAV6‐mCherry) induced a significant increase in locomotor and stereotype behaviours as compared to a single Meth injection (saline/Meth), representing Meth behaviour sensitization. This sensitization response was significantly reduced in mice receiving AAV6‐Meth Ab (AAV6‐MethAb vs. AAV6‐mCherry). *Significant difference, two‐way ANOVA.

A total of 23 mice were used for the locomotor behaviour study. Of these, 12 mice received AAV6‐mCherry or AAV6‐MethAb 14 days before (D − 14, Figure 4A) the induction of Meth sensitization on day 0 (D0). Meth sensitization was induced after repeated administration of Meth (sensitizing dose, 2.5 mg/kg/day, s.c.) for 7 days (days 0–6, see timeline, Figure 4A), as we previously described [24, 25]. Two non‐sensitized control groups (saline/Meth, *n* = 5; saline/saline, *n* = 6) received daily saline injection from days 0 to 6. Locomotor activity was recorded for an hour after Meth (test dose, 1 mg/kg, s.c.; AAV6‐mCherry, AAV6‐MethAb, saline/Meth groups) or saline (saline/saline group) injection on days 7 and 21 (Figure 4B). The pre‐sensitization behaviour was examined on day −1 (D − 1). Four locomotor behaviours (Table 2) were recorded: total distance travelled (TOTDIST), horizontal activity (HACTV), movement time (MOVTIME) and stereotypic movement number (STRCNT).

**TABLE 2:** Significant differences in locomotor behaviours among all groups on D7 and D21.

	Locomotor behaviour (D7 and D21)			
	HACTV	STRCNT	MOVTIME	TOTDIST
AAV MethAb vs. AAV mCherry	*p* = 0.002^a^	0.048	0.013	*p* = 0.045
AAV MethAb vs. saline/Meth	0.061	0.010	0.020	0.022
AAVMethAb vs. saline/saline	*p* < 0.001	0.670	*p* < 0.001	0.001
AAV mCherry vs. saline/Meth	*p* < 0.001	*p* < 0.001	0.028	*p* < 0.001
AAV mCherry vs. saline/saline	*p* < 0.001	*p* < 0.001	*p* < 0.001	*p* < 0.001
Saline/Meth vs. saline/saline	0.063	0.046	0.239	0.340

^a^ Two‐way ANOVA + Fisher LSD test.

Repeated Meth treatment in the AAV6‐mCherry group significantly increased locomotor and stereotype behaviours as compared to a single Meth injection in the saline/Meth group, representing behaviour sensitization (Figure 4, Table 2). AAV6‐MethAb significantly reduced the locomotor/stereotype behaviour in mice receiving repeated Meth injections (AAV6‐MethAb vs. AAV6‐mCherry, Table 2, Figure 4, two‐way ANOVA). A significant difference was found between AAV6‐MethAb and saline/Meth, suggesting that AAV6‐MethAb did not abolish Meth‐mediated locomotor response. No significant difference was found among all groups before Meth sensitization on D − 1 (*p* > 0.05, Table S1).

### 3.5 AAV6‐MethAb Antagonized the Upregulation of TH in Meth‐Sensitized Mice

**FIGURE 5:**
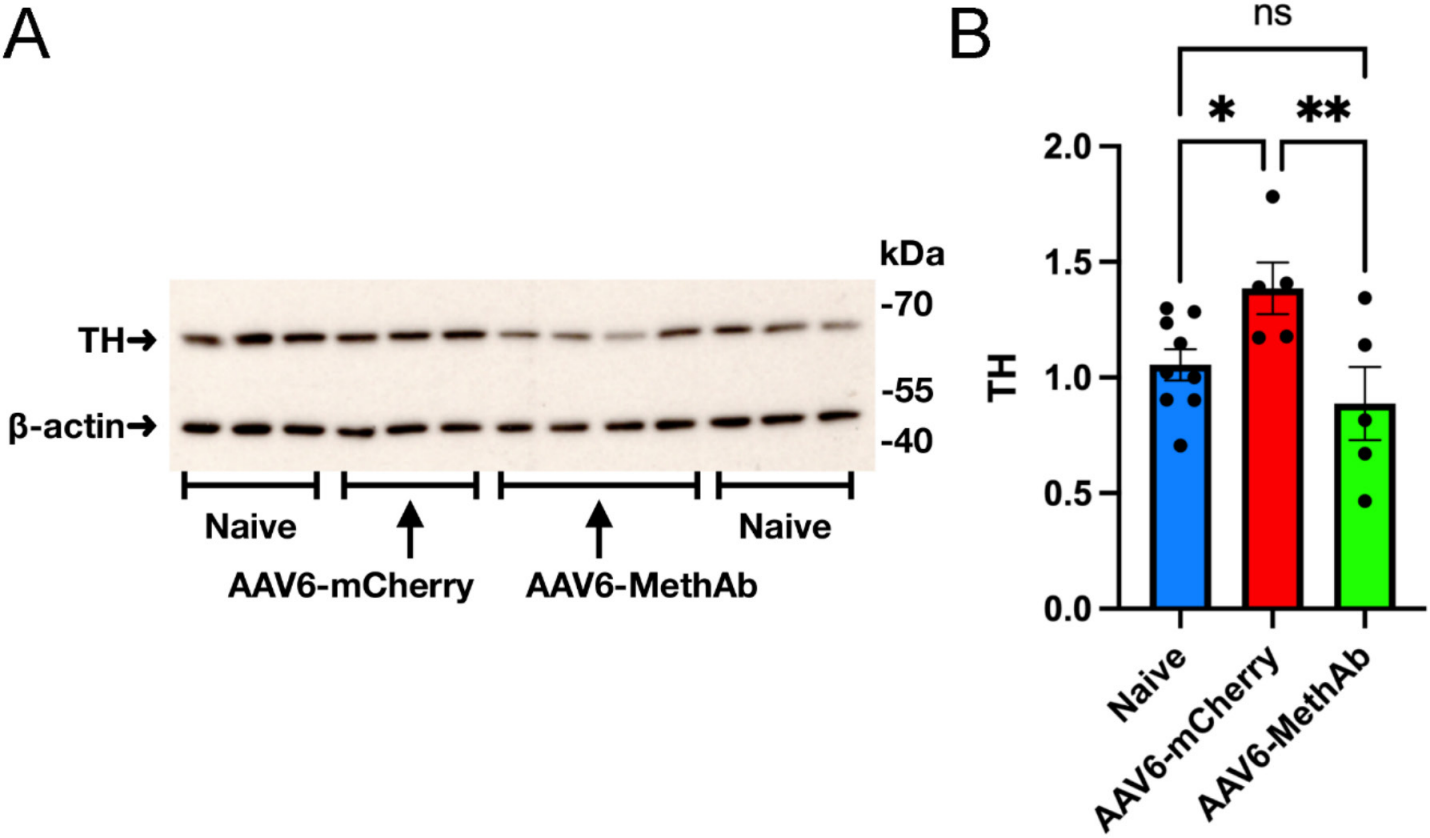
Effects of AAV6‐MethAb on TH expression in Meth‐sensitized mice. Mice were injected with AAV6‐mCherry (*n* = 5) or AAV‐MethAb (*n* = 5) in the NAc on day −14, followed by Meth sensitization from days 0 to 6. Animals were subjected to the Meth‐induced behaviour test on days 7 and 22 and sacrificed on day 23. Naive animals (*n* = 9) were not treated with AAV or Meth. NAc tissues were collected for Western blot analysis. (A) Representative blot demonstrated the expression of TH and β‐actin. (B) The protein levels of TH were quantified and normalized to β‐actin by densitometric analysis. *p < 0.05, **p < 0.01, ns: not significant.

We and others previously demonstrated that repeated administration of Meth‐induced behaviour sensitization was associated with an increase in tyrosine hydroxylase (TH) expression in the NAc [24]. NAc tissue was collected from Meth‐sensitized mice receiving AAV6‐mCherry or AAV6‐MethAb. Western blot analysis showed a significant increase in TH levels in the mice receiving AAV6‐mCherry, compared to the Naïve mice (Figure 5, *p* = 0.036). TH levels were significantly lower in the AAV6‐MethAb mice (Figure 5, *p* = 0.008) than in the AAV6‐mCherry mice. These results suggest that AAV6‐MethAb antagonized the upregulation of TH protein levels in Meth‐sensitized mice.

## 4 Discussion

Chronic or repeated exposure to amphetamine‐type stimulants alters the number of dendrites and the density of dendritic spines of dopaminergic neurons in the NAc [7, 26]. These histological changes of dopaminergic innervation are associated with progressively augmenting behavioural responses to Meth. Similar to these findings, we found that repeated administration of low dose Meth increased dendritic sprouting of primary dopaminergic neuronal cells and enhanced Meth‐mediated locomotor and stereotype behaviour in adult mice. Administration of AAV6‐MethAb effectively transduced MethAb in NAc and reduced Meth‐mediated dopaminergic neuronal sprouting in vitro and significantly mitigated the upregulation of TH in Meth‐sensitized mice. The main finding of this study is that intracerebral administration of AAV6‐MethAb reduced Meth sensitization in chronic Meth‐treated animals. Our study is the first to report AAV‐MethAb‐mediated reduction of Meth sensitization and supports a new gene therapy strategy for Meth abuse.

AAV vectors are considered ideal vehicles for gene delivery in the central nervous system due to their low immunogenicity, non‐pathogenicity and capability to transduce neurons and achieve long‐term transgene expression [27, 28, 29]. Different serotypes of AAV vectors exhibit distinct tissue tropism, likely due to differences in the genome and capsid structure between AAV serotypes [30]. We applied a cross‐packaging strategy to generate several pseudotyped AAV vectors, which package an identical genome within the viral capsids of different serotypes [31, 32]. This strategy allowed us to fairly compare the transduction efficiencies of different serotypes without the influence of the packaged genome and ITR. Among the compared serotypes (AAV1, 2, 5, 6, 7, 8), AAV6 was the most promising vector for transducing rat mesencephalic dopaminergic neurons in vitro. These results suggest that AAV6 is an optimal carrier for expressing MethAb transgene in dopaminergic neurons. We further demonstrated that intracerebral administration of AAV6‐MethAb increased the expression of MethAb in vivo. Several studies have compared the expression levels of GFP gene delivered by AAV vector serotypes 1, 2, 5 [33, 34], 8 [35, 36], 9 and 10 [37] concluded AAV2 as the less effective vector in transducing dopaminergic neurons in the rat substantia nigra. Another in vivo study comparing AAV5, 6, 7 and 8 showed that these serotypes are equally effective [38]. The difference among these studies can be affected by various factors; for example, the product proteins can be trophic or toxic to the target cells and indirectly affect the expression of transgenes and the survival of cells, which warrants further investigation.

In this study, AAV6‐MethAb or AAV6‐mCherry was administered 2 weeks before the administration of Meth in adult mice. We found that Meth‐induced hyperactivity was enhanced after a 7‐day repeated Meth administration, representing Meth sensitization, in the control AAV6‐mCherry mice. Similar to previous studies [39, 40, 41], Meth‐induced hyperactivity was found at 2 weeks after the cessation of 7‐day Meth treatment, indicating the relapse of Meth‐sensitization. Both responses were significantly reduced in the AAV6‐MethAb treated group. These data suggest that CNS‐delivered AAV6‐MethAb antagonized Meth‐mediated behaviour sensitization.

The mesolimbic dopaminergic pathway plays a critical role in the development of Meth behavioural sensitization [42, 43]. Meth sensitization is linked to increases in extracellular dopamine levels in the NAc and striatum [44, 45] and upregulation of BDNF [46] in the NAc. Recently, we reported that a partial lesion of dopaminergic terminals by 6‐hydroxydopamine antagonized Meth‐mediated behavioural sensitization and TH and BDNF expression in the NAc [24]. In this study, we examined the expression of TH in the NAc from Meth‐sensitized mice on day 23. TH expression was significantly upregulated in AAV6‐mCherry animals but not in the mice receiving AAV6‐MethAb, suggesting AAV6‐MethAb normalized TH protein expression in the NAc in Meth‐sensitized mice.

Our previous work characterized the specificity of MethAb with Meth molecules. We demonstrated that intraperitoneal administration of AAV8‐MethAb (2.5 × 10^10^ VGC/mouse) can achieve a long‐term and stable expression of Meth antibodies in the blood. Administration of AAV8‐MethAb attenuated acute (single dose) Meth‐induced hyperactivity and serum Meth level [22]. These data suggested that the MethAb produced after AAV infection bound to Meth molecules in the periphery and prevented the action in the CNS. However, systemic administration demanded a higher viral dose, and it was unclear how efficiently AAV can cross the blood–brain barrier to reach targeted brain areas. In the present study, AAV6 was locally injected into the nucleus accumbens, allowing the expression of the antibody protein in the target brain region involved in the behavioural sensitization, leading to a more effective intervention for Meth addiction. Compared with the peripheral delivery, a much lower dose (a hundred‐fold less) of AAV was needed to neutralize Meth in the brain. Our data support the relatively long‐lasting and highly efficient effect of AAV6‐MethAb against Meth when given intracerebrally.

In this study, we used AAV6 to deliver Meth antibody gene into the brain. This would lead to the localized production of MethAb in the NAc, aiming to reduce the sensitization effect of Meth. As AAV‐MethAb was applied intracerebrally, MethAb was produced mainly locally in NAc, not in the periphery. The centrally expressed MethAb reduced Meth‐mediated sensitization in the brain; it is less efficient to neutralize the adverse side effects (i.e., tachycardia, hypertension, fever, palpitations, shortness of breath, etc.) caused by peripheral Meth. These un‐neutralized peripheral side effects may discourage further Meth use. An indirect example is seen in the treatment of alcohol addiction. Antabuse (disulfiram) is a medication to treat alcohol dependence. When alcohol is consumed with Antabuse, it leads to unpleasant peripheral reactions (nausea, flushing and palpitations, etc.) and reduces alcohol drinking. This strategy, which leverages unpleasant systemic reactions as a deterrent, warrants further investigation in animal models of self‐administration.

Meth addiction remains a significant public health challenge, with limited pharmacological interventions available for treatment [47, 48]. Immunotherapy, particularly antibody‐based strategies, offers a promising approach by reducing Meth bioavailability and neurotoxicity [18, 20]. The present study demonstrates that intracerebral administration of AAV6‐MethAb in the NAc effectively reduces Meth‐induced behavioural sensitization and associated neurochemical changes, highlighting its therapeutic potential. Traditional monoclonal antibody treatments require repeated dosing, limiting their clinical feasibility [49]. AAV‐mediated gene therapy provides a sustained expression of Meth antibodies and long‐term protection after a single administration. In clinical practice, combining AAV6‐MethAb treatment with appropriate counselling or cognitive behavioural therapy may further improve outcomes in drug addicts. The safety of AAV‐based vectors has been evaluated in preclinical and clinical studies [27]. Intracerebral injection of AAV has also been conducted in limited clinical trials [50]. Further studies are still needed to evaluate its immune responses, neuroinflammation, long‐term safety and neurotoxicity. If successfully translated to clinical practice, intracerebral injection of AAV6‐MethAb may serve as a novel therapeutic strategy for preventing Meth relapse and mitigating its neurotoxic effects.

### 4.1 Conclusion

Several factors limit the use of Meth antibody protein directly. For example, in active immunization, a regular and well‐scheduled course of vaccination is required to maintain Meth antibody titres in the blood, as Meth is a weak immunogen [15, 51]. The antibodies induced by active immunization may cross‐react with other molecules structurally similar to Meth. Furthermore, HIV or other immune‐compromised conditions, commonly seen in drug addicts, may yield insufficient antibodies after active immunization [52, 53]. These limitations may be partially overcome by passive immunization. However, repeated administration of purified Meth antibodies is required to reduce Meth responses over an extended period, as would be necessary for chronic Meth users. The high cost of passive immunization would also be a complicating factor likely to reduce compliance in Meth addicts. In contrast, a single and low dose of AAV6‐MethAb, given intracerebrally, efficiently generated Meth antibodies and reduced Meth behaviour sensitization. Our study supports that AAV6‐MethAb is a potential therapeutic agent for chronic Meth abuse.

## Author Contributions

Y.H.C. (manuscript writing, conception, collection and assembly of data); T.W.H. (animal surgery, collection and assembly of data); Y.S.W. (PCR, collection and assembly of data); E.K.B. (cell culture, immunocytochemistry, data analysis); K.J.W. (data analysis); S.J.Y. and Y.W. (conception and design, manuscript writing, administrative support, final approval of manuscript).

## Conflicts of Interest

The authors declare no conflicts of interest.

## Supporting information


**Table S1** No significant difference among all groups before Meth sensitization on D − 1.

## Data Availability

The datasets generated in the current study are available from the corresponding authors on reasonable request (contact: b7508@nhri.edu.tw).
