# Supplementary material for: AAV‐Mediated Expression of Methamphetamine Monoclonal Antibody Attenuates Methamphetamine Behaviour Sensitization in Mice
Source: Addict Biol. 2025 Aug 8;30(8):e70073. doi: 10.1111/adb.70073 (PMC12332764; doi:10.1111/adb.70073)
Supplement: Supplementary file 1 — Table S1 No significant difference among all groups before Meth sensitization on D − 1. [file ADB-30-e70073-s001.docx]

sTable 1. No significant difference among all groups before Meth sensitization on D-1

|  | Locomotor behavior (D-1) | | | |
| --- | --- | --- | --- | --- |
|  | HACTV | STRCNT | MOVE TIME | TOTDIST |
| AAV MethAb  Vs. AAV mCherry | P=0.909 | P=0.902 | P=0.643 | p=0.987 |
| AAV MethAb vs.  saline/Meth | P=0.817 | P=0.912 | P=0.716 | P=0.987 |
| AAVMethAb vs.  Saline/saline | P=0.831 | P=0.935 | P=0.890 | P=0.917 |
| AAV mCherry vs.  saline/Meth | P=0.955 | P=0.791 | P=0.624 | P=0.901 |
| AAV mCherry vs.  saline/saline | P=0.895 | P=0.979 | P=0.752 | P=0.993 |
| Saline/Meth  vs. saline/saline | P=0.699 | P=0.965 | P=0.880 | P=0.995 |

*Two way ANOVA + Fisher LSD test
